# Immunologically-based nutritional status assessment amongst preoperative colorectal cancer patients – does it link to TNM stage

**DOI:** 10.3389/fimmu.2026.1869012

**Published:** 2026-07-10

**Authors:** Karolina Kaźmierczak-Siedlecka, Piotr Wiśniewski, Robert Kucharski, Ewa Stachowska, Wojciech Makarewicz

**Affiliations:** 1 Department of Medical Laboratory Diagnostics – Fahrenheit Biobank BBMRI.pl, Medical University of Gdansk, Gdansk, Poland; 2 International Centre for Cancer Vaccine Science, University of Gdansk, Gdansk, Poland; 3 Neodentica Dentistry Center, Gdansk, Poland; 4 Unit of Surgery with Unit of Oncological Surgery, Specialist Hospital in Koscierzyna, Koscierzyna, Poland; 5 Department of Human Nutrition and Metabolomics, Pomeranian Medical University in Szczecin, Szczecin, Poland; 6 2nd Division of Radiology, Medical University of Gdansk, Gdansk, Poland

**Keywords:** albumin-to-globulin ratio, colorectal cancer, immunonutritional status, prognostic nutritional index, serum albumin, surgery, TNM stage, total lymphocytes count

## Abstract

**Introduction:**

Disease-related malnutrition can develop in colorectal cancer (CRC) patients, negatively affecting postoperative clinical outcomes. Nutritional status should be assessed using standardised tools and laboratory parameters to identify malnourished patients and introduce personalised, targeted dietary interventions based on epigenetics, immunologically-related aspects, and gut microbiome. The primary aim of this study is to analyse the immunonutritional status of CRC patients in the preoperative period. The secondary aim is to assess the link between albumin-to-globulin ratio (AGR), prognostic nutritional index (PNI), and TNM staging.

**Patients and methods:**

This study initially included 21 patients with histopathological confirmation of CRC qualified for surgical resection of the tumour. Immunologically-related nutritional status parameters, i.e., AGR and PNI, were calculated for selected 12 patients. The optimal cut-off value of PNI was determined, allowing patients to be divided into two independent groups: PNI-low and PNI-high. The link between AGR, PNI, and TNM was analysed.

**Results:**

TNM classification of the 12 selected participants revealed that most participants classified as T3 (67%), lymph node metastasis was identified in 50% of patients, and there were no distant metastases. Among selected patients, the analysis of mean values of immunological laboratory tests showed that they were within the normal range, except for lymphocyte levels, which were reduced (22.03 ± 7.31%). The optimal cut-off value was set at 51.74 for PNI; thus, patients were accordingly divided into PNI-low and PNI-high groups (<51.74, 34% of cases; ≥51.74, 66%, respectively). PNI-low was initially associated with more advanced cases (based on T assessment) compared to PNI-high (100% versus 75%; respectively); however, it failed to reach statistical significance (p = 0.515; effect size 0.2889; CI 95%, CI = 0.0110–7.5681). Therefore, this finding should be interpreted with caution. Lymph node metastasis was found more often in the PNI-high group than in the PNI-low group.

**Conclusions:**

Overall concentrations of albumin and total protein were not decreased in CRC patients; however, these parameters allow calculation of AGR and PNI. The link between PNI-low and locally advanced cases of CRC based on T assessment can potentially exist; nevertheless, it should be confirmed with more clinical data. Lymph node metastasis was more often observed in the PNI-high group. This status is also related to higher levels of AGR. A PNI-low value is more related to local than distant pathological processes associated with CRC.

## 1 Introduction

Malnutrition and cancer-related malnutrition in the preoperative period negatively affect postoperative clinical outcomes (1). Colorectal cancer (CRC) patients are at risk of developing malnutrition due to low daily energy and protein intake, gastrointestinal symptoms, local intestinal inflammation, damage to bowel function, malabsorption, disruptions of intestinal epithelial cells, and consequent leaky gut linked to gut microbial imbalance (2–4). Of note, colitis-associated CRC, a major complication of ulcerative colitis with long-term chronic inflammation, contributes to an increased risk of malnutrition (5). It is estimated that approximately 35% of patients undergoing surgical colorectal procedures present with moderate to severe malnutrition in the preoperative period (2). Therefore, nutritional status analysis should be conducted amongst preoperative CRC patients to detect malnourished individuals, introduce appropriate nutritional support, and reduce the risk of postoperative complications.

There are several basic methods used to analyse nutritional status, such as (1): standardised tools (Global Leadership Initiative on Malnutrition – GLIM, Nutritional Risk Screening 2002 – NRS-2002, Patient-Generated Subjective Global Assessment (PG-SGA) (2); anthropometric parameters (Body Mass Index – BMI, unintentional weight loss during the last 3–6 months, composition of body mass regarding the content of fat/muscle mass, and total body water); and (3) laboratory tests (serum concentration of albumin, total protein, total lymphocyte count – TLC) (6, 7). In the retrospective study by Ruan et al. (7) involving CRC patients, both sensitivity and specificity of NRS-2002 with GLIM and PG-SGA were compared. It was observed that NRS-2002 is characterised by superior specificity (NRS-2002: 0.09, GLIM: 0.62, PG-SGA: 0.82) in diagnosing patients without nutritional deficits. Additionally, the prediction of overall survival (OS) at tumour staging was improved by using any of these tools. Notably, survival outcomes are related to nutritional status, which has been reported in a Kapagan et al. cohort study (8) including metastatic CRC patients treated with 5-fluorouracil plus monoclonal antibodies (bevacizumab, cetuximab, or panitumumab) as first-line management. It was demonstrated that malnutrition (defined by Mini Nutritional Assessment-Short Form ≤ 7) is significantly related to decreased OS (p < 0.001).

Due to the fact that multiple trials refer to the nutritional status of CRC patients based on nutritional tools and anthropometric parameters, the current study highlights the problem of immunonutritional status analysis, which may strongly affect clinical outcomes. There are two parameters available to assess immunonutritional status, namely, albumin-to-globulin ratio (AGR) and prognostic nutritional index (PNI) (9). Both albumin and globulins are the main components of serum proteins (10). They are involved in the development of systemic inflammation, representing immune status. Of note, the level of albumin is associated with inflammation and nutritional status, whereas serum globulins are linked to immunity and, similarly, inflammation. Several factors affect the hepatic synthesis of albumin, such as colloid osmotic pressure, inflammation, and nutritional status. Considering globulins, it should be emphasised that they include C-reactive protein, serum amyloid A, and other acute-phase proteins, such as α-1-acid glycoprotein and α-1-antichymotrypsin (11, 12). Therefore, low AGR (i.e., hypoalbuminaemia and hyperglobulinaemia) can be related not only to active inflammation but also to malnutrition (11). The potency of AGR as a prognostic factor for OS and progression-free survival (PFS) has been confirmed (9, 13). PNI reflects both nutritional and immune status. Interestingly, Zhang et al. reported that PNI is a reliable predictor of outcomes amongst patients with gastrointestinal cancer treated with immunotherapy based on immune checkpoint inhibitors (ICIs) (14). Of note, early detection of tumours positively affects survival rates; thus, well-established biomarkers are crucial in this context. The use of fragmentomic features of circulating cell-free mitochondrial DNA is one option recognised as a novel and non-invasive method allowing detection of advanced adenoma/CRC in the early stage (15). In 2026, Shi et al. showed that early detection of CRC is possible using circulating cell-free DNA (cfDNA, miR-129-2) promoter methylation (16). Another promising biomarker is circulating tumour DNA methylation of the *HAND1* (Heart and Neural Crest Derivatives Expressed 1) gene located on chromosome 5q33 (17). Therefore, there is a strong association between CRC diagnosis and epigenetic/genetic factors.

The primary aim of this study is to assess the immunonutritional status of preoperative CRC patients based on immunological laboratory tests (albumin, total protein, and total lymphocyte count), in addition to other parameters, namely, AGR and PNI. The secondary aim is to investigate the association between preoperative AGR/PNI and TNM stage (T – tumour, N – nodes, M – metastasis) amongst these patients.

## 2 Patients and methods

Patients with CRC in the preoperative period were recruited from the Unit of Surgery and the Unit of Oncological Surgery, Specialist Hospital in Koscierzyna, Poland. Inclusion criteria were: age ≥ 18 years, patients with histopathological confirmation of CRC following prior surgical resection of the tumour, and availability of data regarding immunologically-associated laboratory parameters and TNM staging. Exclusion criteria included: age <18 years and CRC patients not qualified for surgery. Patients were classified according to the TNM staging. Immunologically-related laboratory tests, such as serum concentration of albumin, total protein, and TLC, were recorded from patients’ medical records to analyse their immunonutritional status. Based on these parameters, both AGR and PNI were calculated according to the following formulas (10, 14, 18):

1. AGR = albumin/(total protein – albumin)
2. PNI = (10 x serum albumin [g/dL]) + (0.005 x total lymphocyte count [per mm^3^]).

WBCs, neutrophils, lymphocytes, eosinocytes, basocytes, and immature granulocytes were recorded to provide basic immune-associated characteristics of the participants. The normal levels of the analysed laboratory parameters are presented in **Table 1**.

**Table 1:** Normal levels of immunologically associated parameters.

Immunologically associated parameters	Normal levels
WBCs	4–10 [g/dL]
Neutrophils	45–70 [%]
Lymphocytes	25–45 [%]
Eosinocytes	0–5 [%]
Basocytes	0–1 [%]
Immature granulocytes	0–3 [%]
Albumin	35–52 [g/L]
Total protein	60–90 [g/L]
PNI	PNI-high ≥ mean PNI value PNI-low < mean PNI value

WBCs, white blood cells; TLC, total lymphocyte count; AGR, albumin-to-globulin ratio; PNI, prognostic nutritional index.

This study was approved by the Independent Bioethics Committee for Scientific Research at the Medical University of Gdansk (identifier: KB/302-264/2026).

### 2.1 Statistical analysis

Data are presented as the mean ± standard deviation and the range with minimum and maximum values. The immunologically-related nutritional parameters, i.e., AGR and PNI, were calculated according to the above-mentioned formulas using the level of albumin and total protein for AGR, and albumin and TLC for PNI. Overall, receiver operating characteristic (ROC) curve analysis is applied to determine the best cut-off values for these parameters (AGR and PNI). However, in the current study, the value of PNI-low and PNI-high was defined based on statistical methods described by Keskinkilic et al. (18). The optimal cut-off value was defined as the mean PNI of all the study groups, allowing patients to be divided into two independent groups: PNI-low < mean PNI value, and PNI-high ≥ mean PNI value. Statistical significance was determined with a p-value (p < 0.05 was defined as statistically significant). To calculate statistical significance, one-sided and two-sided Fisher’s exact tests were used, as these are suitable for categorical values with a small sample size. A 2 x 2 contingency table was created for T2 and T3/T4 cancer stages associated with low and high values of PNI. Effect size was measured using the odds ratio. The confidence interval (CI) was calculated (95% CI; very wide).

## 3 Results

This study included 21 CRC patients in the preoperative period (**Table 2**). The basic characteristics of all initially included participants were as follows: mean age 68 ± 8.62 years; TNM: predominantly T3 (76.19%), N0 (38%), and M0 (61.9%); the most locally advanced cases were T4aN0M0 and T4aNxM0; one case presented with metastasis (i.e., T3N2aM1b); and 8 cases (38%) had N >0 (T3N2aM1b, T3N2aM0, T3N1aM0 [3 cases], T2N1bM0, T3N1bM0, and T3N2Mx). The mean values of the immunological laboratory parameters were: WBCs 7.43 ± 2.02 g/L, neutrophils 67.50 ± 8.99%, lymphocytes 21.39 ± 7.44%, eosinocytes 3.09 ± 2.74%, basocytes 0.41 ± 0.15%, and immature granulocytes 0.56 ± 0.38%.

**Table 2:** Characteristics of the initially included patients according to the TNM staging and immunological parameters.

Identifier	TNM	WBCs [g/l]	Neutrophils [%]	Lymphocytes [%]	Eosinocytes [%]	Basocytes [%]	Immature granulocytes [%]
**II1b1**	T3N0Mx	5.9	73.4	18.6	3.0	0.5	0.3
IIb2a	T3N2aM1b	10.3	82.5	5.2	0.2	0.4	1.7
IIb3a	T3N0M0	9.5	78.3	15.0	1.1	0.4	1.0
**IIb4a**	T2N0Mx	6.3	62.9	25.4	1.3	0.6	1.2
**IIb5a**	T3N2aM0	6.8	77.3	18.0	1.1	0.3	0.3
IIb6a	T3NxMx	4.4	74.3	14.7	0.4	0.4	0.5
**IIb7a**	T3NxM0	5.9	60.3	32.6	1.1	0.3	0.8
IIb8a	T3N1aM0	7.0	67.0	24.8	0.5	0.1	0.2
**IIb10a**	T3N1aM0	7.8	64.4	23.2	3.0	0.3	0.2
**IIb11a**	T3N1aM0	6.0	73.4	15.5	2.9	0.7	0.8
**IIb12a**	T4aN0M0	6.6	54.4	28.6	7.0	0.7	0.3
IIb13a	T3NxMx	8.6	67.8	19.6	7.3	0.3	0.9
**IIb14a**	T2N1bM0	8.2	61.3	25.5	4.5	0.4	0.6
IIb15a	T3N0Mx	5.6	58.4	30.4	4.1	0.6	0.1
IIb16a	T2N0M0	10.4	47.4	29.6	11.2	0.4	0.3
IIb17a	T3N0M0	5.8	69.0	21.9	2.4	0.2	0.3
**IIb18a**	T3N1b M0	8.5	60.0	30.8	3.1	0.5	0.4
IIb19a	T3N0Mx	4.05	62.5	23.5	1.6	0.4	0.3
**IIb20a**	T3NxM0	12.1	75.7	11.5	6.3	0.3	0.4
**IIb21a**	T4aNxM0	9.2	81.9	8.4	1.7	0.4	0.4
**IIb22a**	T3N2Mx	7.0	65.3	26.3	1.1	0.4	0.7

TNM (T, tumour; N, nodes; M, metastasis), WBCs, white blood cells.

Bold values indicates patients with complete data allowing to conduct further analysis.

Among the initially included patients (n = 21), 12 participants were selected (**Table 3**) because complete data were available for the above-mentioned immunological laboratory parameters and the concentrations of serum albumin and total protein, allowing calculation of the immunologically-based parameters AGR and PNI.

**Table 3:** Characteristics of patients with complete immunonutritional parameter data.

Identifier	TNM	Albumin [g/l]	Total protein [g/l]	TLC	AGR	PNI
II1b1	T3N0Mx	41	68	1097.4	1.52	46.49
IIb4a	T2N0Mx	44	66	1600.2	2.0	52.0
IIb5a	T3N2aM0	48	69	1224.0	2.29	54.12
IIb7a	T3NxM0	43	65	1923.4	1.95	52.62
IIb10a	T3N1aM0	45	70	1809.6	1.8	54.05
IIb11a	T3N1aM0	47	77	930.0	1.57	51.65
IIb12a	T4aN0M0	43	65	1887.6	1.95	52.44
IIb14a	T2N1bM0	46	69	2091.0	2.0	56.46
IIb18a	T3N1bM0	42	63	2618.0	2.0	55.09
IIb20a	T3NxM0	44	65	1391.5	2.1	50.96
IIb21a	T4aNxM0	39	63	772.8	1.63	42.86
IIb22a	T3N2Mx	43	67	1841.0	1.79	52.21

TNM (T, tumour; N, nodes; M, metastasis), TLC, total lymphocytes count; AGR, albumin-to-globulin ratio; PNI – prognostic nutritional index.

Considering the TNM stage of the selected 12 participants, the majority were assessed as T3 (67%), six cases were identified as N >0 (50%), and none had M >0. The basic immunological laboratory characteristics were as follows: mean WBCs 7.53 ± 1.73 g/L, neutrophils 67.53 ± 8.13%, lymphocytes 22.03 ± 7.31%, eosinocytes 3.01 ± 1.93%, basocytes 0.45 ± 0.14%, and immature granulocytes 0.53 ± 0.28%.

The levels of serum albumin and total protein amongst preoperative patients with CRC (n = 12) were: albumin, range 39–48 g/L, mean 43.75 ± 2.42 g/L; and total protein, range 63–77 g/L, mean 67.25 ± 3.68 g/L. All patients were characterised by a normal level of serum albumin and total protein.

According to the previously mentioned formulas, both parameters, i.e., AGR and PNI, were calculated for these 12 participants (mean AGR 1.88 ± 0.22 and mean PNI 51.74 ± 3.57). The optimal cut-off value was defined as the mean PNI value of all patients with complete immunological data, which was 51.74. Based on this value, patients were divided into two independent groups: PNI-low <51.74 and PNI-high ≥51.74. The first group, characterised by PNI-low <51.74, included 34% of patients. The second comprised 66% of participants with PNI-high. Among patients with PNI-low, there was the following TNM assessment: T3N0Mx, T3N1aM0, T3NxM0, and T4aNxM0. Thus, T3 was observed in 75% of cases, whereas T4 was observed in 25%. N >0 was observed in one case (N1a, 25%). No cases with M >0 were detected, and in one patient the M stage could not be assessed. TNM stages amongst PNI-high patients were as follows: T2N0Mx, T3N2aM0, T3NxM0, T3N1aM0, T4aN0M0, T2N1bM0, T3N1bM0, and T3N2Mx. Therefore, the majority of patients were assessed as T3 (62.5%), followed by T2 (25%) and T4 (12.5%). N>0 was observed in 50% of patients with PNI-high. Considering T3 and T4 together as advanced T, 100% of patients in the PNI-low group had advanced T stage (T3 + T4), whereas in the PNI-high group it was 75% (T3 + T4). However, this difference was not statistically significant (p = 0.515; effect size = 0.2889; CI = 0.0110, 7.5681). The mean value of AGR for the PNI-high group was 1.9725, whereas for the PNI-low group it was 1.705. Therefore, patients with status PNI-high were characterised by a higher AGR value.

## 4 Discussion 

The aetiopathogenesis of CRC-related malnutrition is multifactorial, involving low consumption of energy/nutrients together with aspects associated with local intestinal inflammation. Of note, malnutrition impairs immune function (19). Additionally, there is a link between nutritional status (including malnutrition), the gut microbiome, and tumourigenic pathways, which has been confirmed by Chao et al. (20). The overall approach to CRC is based on the TNM staging system (T – the infiltration depth of the primary tumour, N – lymph node metastasis, M – distant metastases) (11, 21). In the current study, patients with complete data were predominantly classified as T3 (67%). Lymph node metastasis was detected in 50% of cases, whereas distant metastases were not found. Analysis of the mean values of the immunological laboratory parameters (WBCs, neutrophils, lymphocytes, eosinocytes, basocytes, and immature granulocytes) revealed that they were within the normal range, except the level of lymphocytes, which was decreased (22.03 ± 7.31%; normal range 25%–45%). The reduction in lymphocyte count affects the PNI value. All patients had normal concentrations of serum albumin and total protein (minimum values 39 g/L and 63 g/L; maximum values 48 g/L and 77 g/L; mean values 43.75 g/L and 67.25 g/L; standard deviations 2.42 and 3.68, respectively). Of note, it is estimated that albumin accounts for approximately 50% of total plasma protein, making it the predominant protein (22). A decrease in serum albumin concentration is associated with increased levels of pro-inflammatory mediators, including IL-1, IL-6, and TNF-α (23). Hypoproteinaemia and hypoalbuminaemia are linked to impaired immune function, promoting an inflammatory environment by, amongst other mechanisms, stimulating the release of pro-inflammatory cytokines and consequently resulting in a poor prognosis (24). The results obtained in the current study demonstrate that serum albumin does not by itself reflect nutritional status. However, its level is used to calculate multiple parameters, including AGR, PNI, CAR (C-reactive protein-to-albumin ratio), and the CALLY (C-reactive protein – albumin – lymphocyte) index (25, 26). Albumin is also included into the system regarding *RAS* gene mutations, i.e., ALRI (albumin – total lymphocyte count – *RAS* index) (27). The parameters AGR and PNI were analysed in the current study and are discussed below.

AGR is a haematological marker reflecting systemic inflammation and nutritional status. It is strongly associated with postoperative outcomes in CRC (28, 29). PNI value reflects the combination of nutritional status (serum albumin) and immunological aspect (total lymphocyte count), thereby representing immunonutritional status. A low level of PNI is related to shorter OS and poorer postoperative outcomes (15, 30). In the current study, the association between PNI and TNM stage was analysed. The result of T assessment indicated that PNI-low could be associated with more advanced cases of CRC tumour compared to PNI-high (100% versus 75%, respectively). However, this association was not confirmed due to lack of statistical significance (p = 0.515). When considering N >0 (the involvement of lymph nodes metastasis), this was observed more frequently in the PNI-high group than in the PNI-low group (50% versus 25%, respectively). In both groups, distant metastasis was not detected. Despite these initial results, no conclusion can be drawn regarding an association between PNI-low and locally advanced disease. This relationship requires confirmation by more clinical data. This parameter, especially in perioperative period is significant because it affects postoperative recovery/outcome. Recently, in 2025, Bu et al. (31) reported that PNI ≤48.78 (with a cut-off value of PNI 48.78 based on the highest Youden index) and laparotomy were defined as risk factors for short-term postoperative complications.

### 4.1 How to improve immunonutritional status?

A nutritional strategy that modifies the functioning of the immune system is immunonutrition, defined as a diet enriched with compounds possessing immunomodulatory properties. Immunonutrition in oncology primarily includes arginine, glutamine, nucleotides, omega-3 fatty acids, and specific modulators of the gut microbiome/gastrointestinal immunity/gut microbiome–immune system axis, including probiotics, next-generation probiotics, and postbiotics. In a systematic review and network meta-analysis (32) including 34 studies with 2,841 participants, it was noted that glutamine, arginine, and omega-3 fatty acids affect the levels of immunological mediators by decreasing pro-inflammatory TNF-α and IL-6. Of note, the combination of arginine and omega-3 fatty acids was more effective at reducing IL-6, whereas glutamine was superior in decreasing TNF-α. Regarding probiotics, it should be emphasised that they were found to be effective in decreasing the incidence of pneumonia. The administration of probiotics during the perioperative period should be considered to, amongst other benefits, reduce the incidence of postoperative complications. During the preoperative period, arginine is particularly important due to its role in supporting the wound-healing process.

There is a link between gut microbiome and immune system with bidirectional interactions, which means that microorganisms residing in the gastrointestinal tract affect the activity of the immune system and vice versa. Therefore, this relationship can be considered the gut microbiome–immune system axis. Recently, in 2025, Kaźmierczak-Siedlecka et al. (33) reported that an enteral immunodiet does not affect, per se, the gut microbiome in either gastric cancer or CRC patients during the preoperative period. Nevertheless, the identification of several taxa with a nominal p-value <0.05 (*Bilophila*, *Clostridium sensu stricto 1*, *Coprobacter*, *CAG-56*, *Holdemania*, *Ruminococcus*, *Fusicatenibacter*, and *[Eubacterium] eligens*) suggests the potential of immunonutrition to alter the composition of the microbiome and its activity.

Immunodiets and therapeutic methods of gut microbiome modulation seem to be promising strategies for surgical CRC patients. To optimise the efficiency of immunonutrition, it should be introduced as part of a personalised nutritional approach. Therefore, it is necessary to assess not only nutritional status but also immunonutritional status. It is also recommended to analyse the gut microbiome due to its unique individual signature and strong connection with the immune system, recognised as the intestinal microbiome–immune system axis. Epigenetic aspects are also important in designing a personalised nutritional approach.

### 4.2 Limitations and future directions

There are several possible limitations of this study, as follows:

- Relatively small sample size (n = 21), limiting statistical power.

Of note, the sample size is excessively small; therefore, it significantly influences statistical power and raises the risk of false negatives. Nevertheless, these findings should be regarded as preliminary and serve to explore the problem of CRC-related malnutrition in the context of immunological aspects in preoperative patients. This study provides the basis for a subsequent trial investigating the impact of an immunomodulatory product (containing probiotics) on immunonutritional status during the perioperative period.

- Final analysis.

Only 12 of the initial 21 patients were included in the final analysis; thus, 9 participants were excluded. These 12 patients were selected because complete data were available for the immunological parameters required to calculate the immunologically based parameters (AGR and PNI). The concentrations of albumin and total protein were required to calculate AGR, whilst serum albumin and total lymphocyte count were required to calculate PNI. These parameters were not found in the medical records of 9 patients; therefore, they were excluded due to incomplete data.

- No data regarding the level of prealbumin.

The current study evaluated the concentration of serum albumin, which has a half-life of approximately 21 days. To obtain a stable state, 3 months are required. In contrast, the half-life of prealbumin is approximately 2–3 days; thus, it reflects the synthesis of protein more quickly/stably (34). Its decreased level is observed in malnutrition. Therefore, it could be beneficial to analyse not only the level of albumin and total protein, but also prealbumin.

## 5 Conclusions

Assessment of immunologically based nutritional status is required in CRC patients during the preoperative period. There are two independent parameters, PNI and AGR, which reflect both nutritional status and immunological aspects, thus allowing the assessment of immunonutritional status based on laboratory tests measuring the level of albumin, total protein, and TLC. The majority of CRC patients were classified as having high PNI (66%). Considering TNM assessment with T background, all patients in the low PNI group had T3 + T4 (100% versus 75% in the PNI-high group). This suggests that PNI-low status was associated with locally advanced cases; nevertheless, its significance is low due to failing statistical power (p = 0.515). Patients with high PNI were characterised by a higher level of AGR. A potential association between immunonutritional status and TNM, especially in the case of T assessment, may exist; however, this requires confirmation with more clinical data.

## Data Availability

The datasets presented in this study can be found in online repositories. The names of the repository/repositories and accession number(s) can be found in the article/supplementary material.
